# Inhibitory Effect of Purpurogallin on Osteoclast Differentiation In Vitro through the Downregulation of c-Fos and NFATc1

**DOI:** 10.3390/ijms19020601

**Published:** 2018-02-17

**Authors:** Kiryeong Kim, Tae Hoon Kim, Hye Jung Ihn, Jung Eun Kim, Je-Yong Choi, Hong-In Shin, Eui Kyun Park

**Affiliations:** 1 Department of Oral Pathology and Regenerative Medicine, School of Dentistry, IHBR, Kyungpook National University, Daegu 41940, Korea; kileyong93@daum.net (K.K.); hjpihn@hanmail.net (H.J.I.); hishin@knu.ac.kr (H.-I.S.); 2 Department of Food Science and Biotechnology, Daegu University, Gyeongsan 38453, Korea; skyey7@daegu.ac.kr; 3 Department of Molecular Medicine, School of Medicine, Kyungpook National University, Daegu 41944, Korea; kjeun@knu.ac.kr; 4 Department of Biochemistry and Cell Biology, School of Medicine, Kyungpook National University, Daegu 41944, Korea; jechoi@knu.ac.kr

**Keywords:** purpurogallin, RANKL, osteoclastogenesis, Blimp1

## Abstract

Purpurogallin, a benzotropolone-containing natural compound, has been reported to exhibit numerous biological and pharmacological functions, such as antioxidant, anticancer, and anti-inflammatory effects. In this study, we enzymatically synthesized purpurogallin from pyrogallol and investigated its role in receptor activator of nuclear factor-κB ligand (RANKL)-induced osteoclastogenesis. Purpurogallin attenuated the formation of multinucleated tartrate-resistant acid phosphatase (TRAP)-positive osteoclasts from bone marrow macrophages (BMMs) without causing cytotoxicity, and suppressed upregulation of osteoclast-specific markers, including TRAP (*Acp5*), cathepsin K (*Ctsk*), and dendritic cell-specific transmembrane protein (*Dcstamp*). However, purpurogallin did not affect the bone resorbing function of mature osteoclasts evident by the resorption pit assay. Activation of mitogen-activated protein kinases, Akt and IkB pathways in RANK signaling were not altered by purpurogallin, whereas the expression of c-Fos and NFATc1, key transcriptional regulators in osteoclastogenesis, was dramatically inhibited by purpurogallin. Purpurogallin also significantly reduced the expression level of B lymphocyte-induced maturation protein-1 (Blimp1) gene (*Prdm1*). Further, downregulation of Blimp1 led to forced expression of anti-osteoclastogenic genes, including interferon regulatory factor-8 (*Irf8*) and B-cell lymphoma 6 (*Bcl6*) genes. Taken together, our data suggested that purpurogallin inhibits osteoclast differentiation via downregulation of c-Fos and NFATc1.

## 1. Introduction

The interaction between bone-forming osteoblasts and bone-resorbing osteoclasts is crucial for maintaining bone homeostasis [1]. Disruption of this homeostasis caused by a variety of pathophysiological conditions, such as hormonal perturbation, inflammation, aging, stress, and dietary habits, leads to skeletal diseases. In particular, an abnormal increase in osteoclast formation and activation results in bone loss associated with osteoporosis and osteolytic diseases [2]. Thus, suppression of osteoclast differentiation or blocking osteoclast function can be an effective way for treating the skeletal diseases associated with abnormally enhanced activation of osteoclasts [3,4]. Bisphosphonate, the most commonly prescribed osteoporosis drug, has been reported to inhibit bone resorption by inducing osteoclast apoptosis and inhibiting osteoclast function [5,6]. However, bisphosphonate can cause severe side effects, such as hypocalcemia and osteonecrosis in the jaw [7,8,9]. Therefore, alternative therapies are necessary to avoid such side effects. Natural compounds from medicinal plants and foods have drawn attention for developing more effective and safer therapeutic agents for skeletal diseases.

Purpurogallin, a benzotropolone-containing compound, occurs naturally in the nut gall of *Quercus* spp. [10,11]. It has been reported to inhibit the activity of enzymes, including glutathione S-transferase, xanthine oxidase, and catechol *O*-methyltransferase [12,13,14]. In addition, purpurogallin has been shown to have peroxyl radical scavenging activity [15]. It protects ventricular myocytes and aortic endothelial cells from peroxyl radicals [16,17]. It also protects hemolysis of red blood cells induced by peroxyl radicals [18]. Inflammatory responses are also modulated by purpurogallin. Purpurogallin reduces the production of pro-inflammatory cytokines, such as tumor necrosis factor-α (TNF-α) and interleukin-1 (IL-1), in lipopolysaccharide (LPS)-stimulated BV2 cells. The inhibitory effect of purpurogallin is mediated by inhibiting mitogen-activated protein kinases (MAPKs) and nuclear factor-kappa B (NF-κB) signaling pathways [19].

MAPKs and NF-κB signaling pathways are critical for osteoclast differentiation and function, and are mediated by a variety of cytokines, such as receptor activator of nuclear factor kappa-B ligand (RANKL), IL-1, and TNF-α [20]. In particular, RANKL interaction with the receptor activator of nuclear factor kappa-B (RANK) activates multiple critical signaling pathways, leading to the expression of genes involved in osteoclast differentiation and function. Inhibition of the extracellular signal-regulated kinase (ERK), p38, and c-Jun N-terminal kinase (JNK) induces suppression of RANKL-induced osteoclast differentiation [21]. Phosphoinositide 3-kinase and Akt/PKB are also important for osteoclast differentiation and function [22,23]. Through these signaling pathways, transcription factors such as c-Fos, NF-κB, and nuclear factor of activated T-cell (NFATc1) are activated and regulate the expression of genes necessary for osteoclast differentiation [24].

In the present study, we investigated whether purpurogallin could inhibit RANKL-induced osteoclast differentiation of bone marrow macrophages. We also examined the molecular mechanisms underlying the inhibitory effect of purpurogallin during osteoclast differentiation.

## 2. Results

### 2.1. Purpurogallin Suppresses Receptor Activator of Nuclear Factor Kappa-B Ligand (RANKL)-Mediated Osteoclast Differentiation of Bone Marrow Macrophages (BMMs)

**Figure 1:**
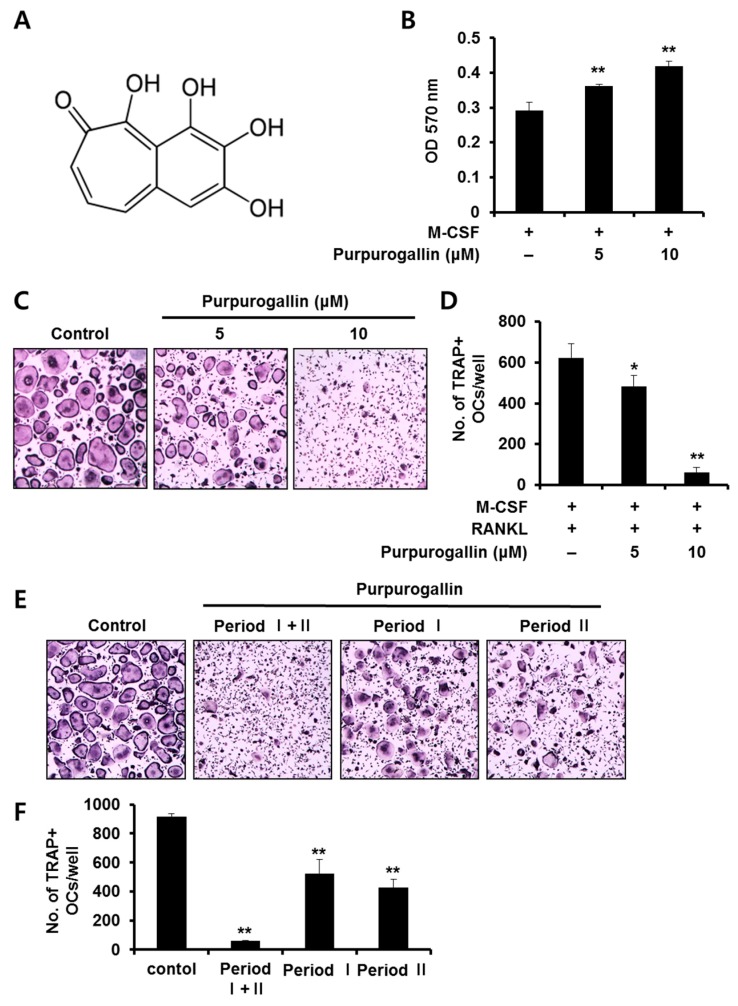
Purpurogallin inhibits receptor activator of nuclear factor kappa-B ligand (RANKL)-induced osteoclast differentiation. (**A**) Chemical structure of purpurogallin; (**B**) BMMs were incubated with M-CSF (10 ng/mL) in the presence of 0, 5, or 10 μM purpurogallin for 3 days. Cell viability was assessed using the MTT assay. ** *p* < 0.01 versus vehicle-treated control; (**C**) BMMs were incubated with M-CSF (10 ng/mL) and RANKL (20 ng/mL) in the presence of 0, 5, or 10 μM purpurogallin for 4 days. The cells were stained for TRAP. Magnification 50×; (**D**) TRAP-positive cells with more than three nuclei were scored. * *p* < 0.05, ** *p* < 0.01 versus vehicle-treated control; (**E**) BMMs were cultured with M-CSF (10 ng/mL) and RANKL (20 ng/mL) in the presence or absence of purpurogallin (10 μM). Purpurogallin was added from day 0 to day 4 (period I + II), from day 0 to day 2 (period I), or from day 2 to day 4 (period II). Osteoclast formation was assessed by TRAP staining. Magnification 50×; (**F**) TRAP-positive multinucleated cells with ≥ 3 nuclei were counted. ** *p* < 0.01 versus vehicle-treated control.

To assess the effect of purpurogallin on RANKL-mediated osteoclast differentiation, BMMs stimulated with M-CSF (10 ng/mL) and RANKL (20 ng/mL) were incubated in the presence or absence of 5 or 10 μM purpurogallin. After 4 days, the cells were fixed and stained with TRAP. Osteoclasts were evidenced by the appearance of multinuclear TRAP-positive cells. The formation of TRAP-positive MNCs was effectively inhibited by treatment with purpurogallin (Figure 1C). The number of TRAP-positive MNCs decreased in a concentration-dependent manner (Figure 1D). To examine the effect of purpurogallin on cell viability, BMMs were cultured with M-CSF (10 ng/mL) in the presence or absence of purpurogallin (5 or 10 μM) for 3 days. The cytotoxicity of osteoclast precursors was evaluated using the MTT assay. The results showed that purpurogallin did not exhibit cytotoxic effects on BMMs at the tested doses (Figure 1B).

### 2.2. Purpurogallin Inhibits both Early and Late Stages of Osteoclast Differentiation

To examine the stage-specific inhibitory effect of purpurogallin on osteoclast differentiation, it was added at an early stage (Period I: days 0–2), a late stage (Period II: day 2–4), or all stages (Period I + II) during osteoclast differentiation. When purpurogallin (10 μM) was added at all stages, osteoclast formation was dramatically inhibited (Figure 1E,F). However, the inhibitory effect of purpurogallin on osteoclast formation was lower when the drug was added at the early or late stage than during the whole period (Figure 1E,F). These results demonstrated that purpurogallin exerts its inhibitory action efficiently when treated at all stages of osteoclast differentiation.

### 2.3. Purpurogallin Downregulates the Expression of Key Regulatory Factors and Osteoclast Marker Genes

**Figure 2:**
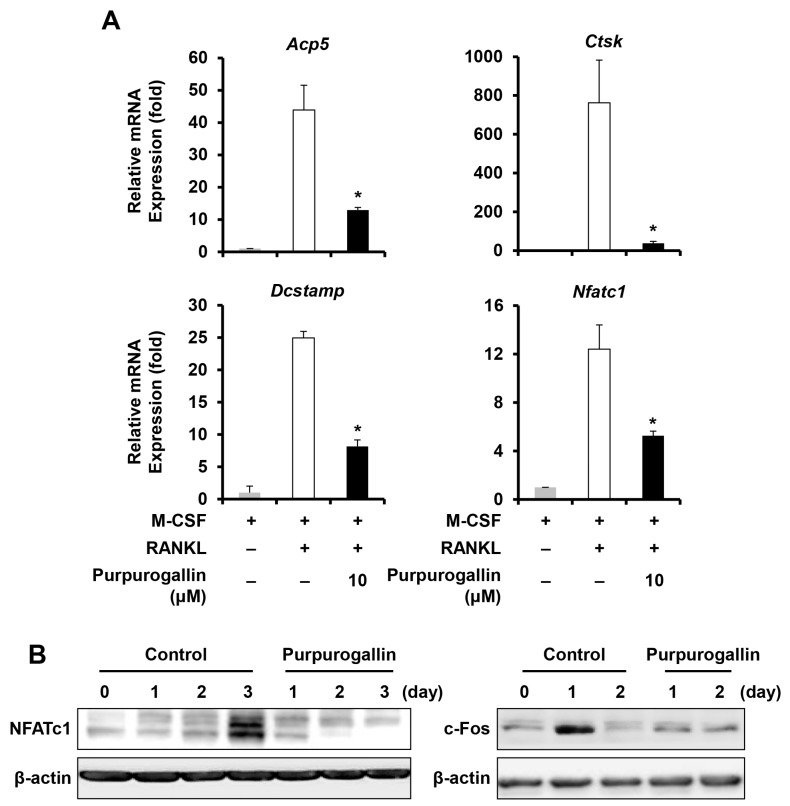
Purpurogallin treatment ameliorates the expression of osteoclast-specific markers during osteoclastogenesis. (**A**) BMMs were cultured in an osteoclastogenic medium with vehicle or purpurogallin (10 μM) for 4 days. The mRNA expression of TRAP (*Acp5*), cathepsin K (*Ctsk*), DC-STAMP (*Dcstamp*), and NFATc1 (*Nfatc1*) genes was evaluated using real-time PCR. * *p* < 0.05; (**B**) BMMs were cultured in an osteoclastogenic medium in the absence or presence of 10 μM purpurogallin for the indicated time period. The protein expression of NFATc1 and c-Fos was determined using western blotting.

To further confirm that purpurogallin inhibits osteoclast differentiation, we examined mRNA expression levels of osteoclast-specific marker genes, such as tartrate-resistant acid phosphatase (*Acp5*), cathepsin K (*Ctsk*), and dendritic cell-specific transmembrane protein (*Dcstamp*), after purpurogallin treatment. Real-time PCR analysis demonstrated that the expression of osteoclastic marker genes was upregulated by stimulation of RANKL, whereas purpurogallin downregulated the expression of the osteoclast-specific genes (Figure 2A). In addition, to understand the inhibitory mode of action of purpurogallin on osteoclast differentiation, the expression of c-Fos and NFATc1, the key transcription factors, were examined. The results showed that the mRNA level of *Nfatc1* and the protein level of NFATc1 and c-Fos were dramatically decreased following purpurogallin treatment (Figure 2A,B).

### 2.4. Purpurogallin Inhibits the Formation of Actin Rings

**Figure 3:**
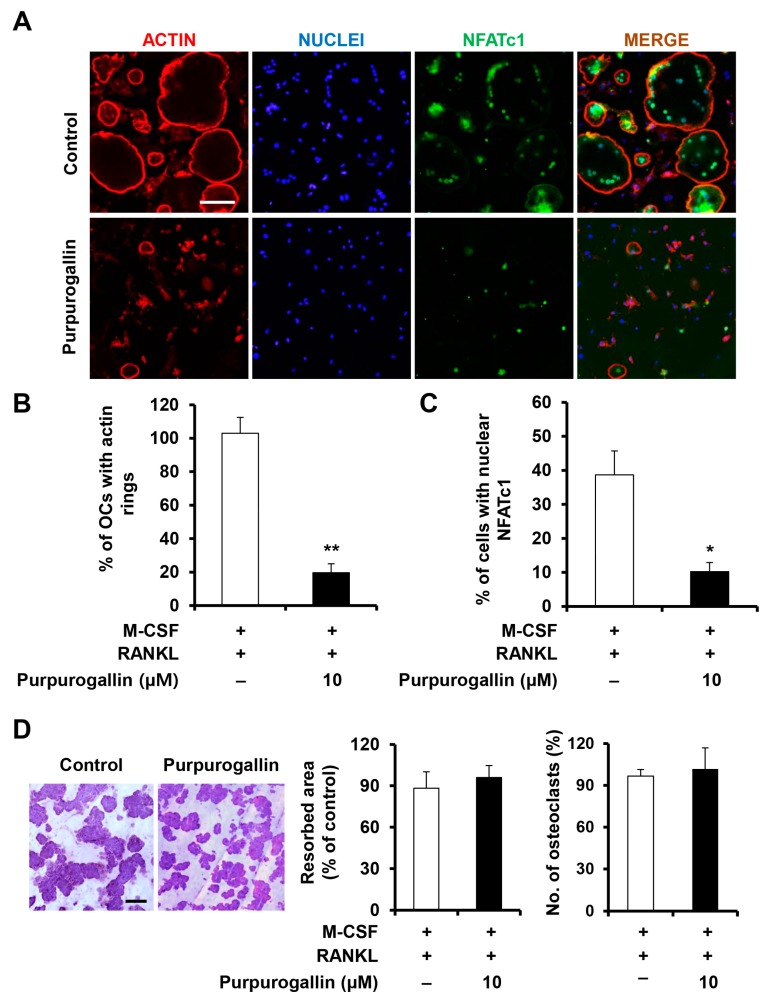
Purpurogallin inhibits the formation of actin rings, but does not suppress the formation of resorption pits. (**A**) BMMs seeded onto glass coverslips were cultured in an osteoclastogenic medium containing M-CSF (10 ng/mL) and RANKL (20 ng/mL) with or without purpurogallin (10 μM). The cells were stained with the anti-NFATc1 antibody, and actin rings and nuclei were stained with rhodamine-conjugated phalloidin and DAPI, respectively. Scale bar, 100 μm; (**B**) The number of actin rings and (**C**) the number of cells with nuclear NFATc1 were analyzed. * *p* < 0.05, ** *p* < 0.01. (**D**) BMMs were plated onto bone slices and incubated with M-CSF (10 ng/mL) and RANKL (20 ng/mL) for 3 days to induce osteoclast differentiation. Next, the cells were treated with or without purpurogallin (10 μM) for 2 days. Multinucleated osteoclasts were counted, and resorption pits were visualized by hematoxylin staining.

Mature osteoclasts secrete several proteinases and pump out protons to resorb the bone, and they also form an actin ring structure to confine the acidic resorbing microenvironment [25]. As shown in Figure 3A, mature osteoclasts form circular actin rings in the periphery of cells. However, purpurogallin treatment suppressed the formation of actin rings (Figure 3A,B). To further investigate whether purpurogallin affected the activity of mature osteoclasts, BMMs were incubated in an osteoclastogenic medium for 3 days to form multinucleated osteoclasts, and treated with purpurogallin for an additional 2 days. Compared to the control, treatment with purpurogallin showed no effect on resorption pit formation by osteoclasts (Figure 3D). To normalize resorption pit formation, the number of osteoclasts was counted, and the numbers of multinucleated osteoclasts were not decreased by purpurogallin (Figure 3D).

### 2.5. Purpurogallin Induces the Expression of Negative Regulating Factors of RANKL-Induced Osteoclast Differentiation

**Figure 4:**
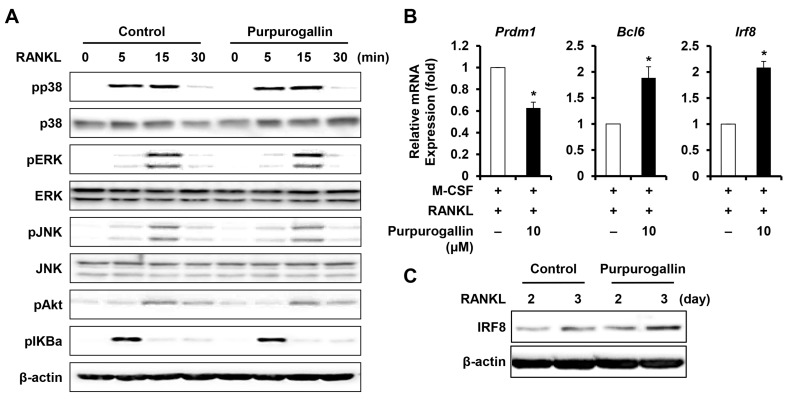
Purpurogallin inhibits the suppression of negative mediators of RANKL-induced osteoclast differentiation. (**A**) BMMs were incubated in a serum-free medium for 5 h, and subsequently pretreated with purpurogallin (10 μM) or vehicle for 1 h before RANKL (50 ng/mL) stimulation. Phosphorylation of p38, ERK, JNK, Akt, and IκBα was assessed using western blotting; (**B**) BMMs were incubated in an osteoclastogenic medium with or without purpurogallin for 4 days. The mRNA expression of *Prdm1*, *Bcl6*, and *Irf8* was analyzed using real-time RT-PCR. * *p* < 0.05; (**C**) BMMs were incubated in an osteoclastogenic medium with or without 10 μM purpurogallin for the indicated time period. IRF8 protein expression was analyzed using western blotting.

We next examined whether purpurogallin affected the RANK signaling pathways, such as MAPK and NF-κB pathways. BMMs were pretreated with purpurogallin (10 μM) or vehicle for 1 h before RANKL (50 ng/mL) stimulation. Purpurogallin did not affect the RANKL-induced phosphorylation of p38, ERK, JNK, AKT, and IκBα (Figure 4A). These results suggested that purpurogallin inhibits osteoclast differentiation through other regulatory mechanisms. We, therefore, examined the expression of negative regulators of osteoclast differentiation. RANK signaling suppresses the expression of negative regulators, such as interferon regulatory factor-8 (IRF8) and B-cell lymphoma 6 (Bcl6) through the upregulation of Blimp1, a transcriptional repressor of Bcl6 and IRF8 [26]. We used real-time PCR to assess the expression of negative mediators of osteoclast differentiation. The mRNA expression of *Irf8* and *Bcl6* was upregulated by purpurogallin treatment (Figure 4B) and the protein level of IRF8 also increased (Figure 4C). In addition, we confirmed that mRNA expression of *Prdm1* was downregulated (Figure 4B).

### 2.6. Purpurogallin Strongly Inhibits Osteoclast Differentiation more than Pyrogallol

**Figure 5:**
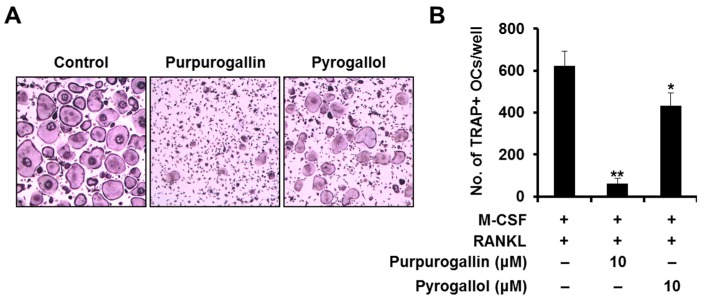
Purpurogallin inhibits osteoclast differentiation more effectively than pyrogallol. (**A**) BMMs were cultured with M-CSF (10 ng/mL) and RANKL (20 ng/mL) in the presence of purpurogallin or pyrogallol (10 μM). The cells were stained for TRAP; (**B**) TRAP-positive cells with more than three nuclei were scored. * *p* < 0.05, ** *p* < 0.01 versus vehicle-treated control.

To examine whether the inhibitory effect of purpurogallin on RANKL-induced osteoclast differentiation was comparable with that of pyrogallol, a precursor of purpurogallin, we treated with M-CSF (10 ng/mL) and RANKL (20 ng/mL) in the presence of purpurogallin or pyrogallol (10 μM). When purpurogallin was added at a concentration of 10 μM, osteoclast differentiation was almost completely inhibited. In contrast, when pyrogallol was added at the same concentration as purpurogallin, osteoclast formation was only partially inhibited (Figure 5A). Compared to control, the number of TRAP-positive osteoclasts also dramatically decreased following purpurogallin treatment (90.2%), but not following pyrogallol treatment (30.5%) (Figure 5B).

## 3. Discussion

As the demand for development of safer alternative agents for the treatment and prevention of osteolytic bone diseases increases, natural compounds and their derivatives are being increasingly focused upon due to their various beneficial effects. Purpurogallin occurs naturally in oak trees infested with hymenopterous insects, and can also be synthesized by the chemical oxidation of pyrogallol using peroxidase [27]. Under physiological conditions (pH 7.4), pyrogallol is unstable and rapidly converts to purpurogallin [13]. Honda et al. reported that purpurogallin exhibits higher affinity for xanthine oxidase (XO) than pyrogallol and plays an important role in enhancing the XO inhibitory effect of pyrogallol [13]. In the present study, we also observed that purpurogallin inhibited osteoclast formation more efficiently than pyrogallol (Figure 5).

To explore the effect of purpurogallin on osteoclast differentiation, we synthesized it from pyrogallol. Purpurogallin significantly inhibited osteoclast differentiation in a dose-dependent manner without causing cytotoxicity (Figure 1). Consistently, purpurogallin suppressed the expression of *Acp5*, *Ctsk*, and *Dcstamp*, which are the marker genes of osteoclast differentiation (Figure 2A), indicating that purpurogallin has an inhibitory effect on osteoclast formation.

Cell-to-cell fusion to form multinucleated osteoclasts is associated with the formation of actin rings. The actin ring is tightly bound to the bone matrix. Subsequently, the actin ring confines a proton gradient which is necessary to resorb the bone [28]. Purpurogallin treatment significantly inhibited actin ring formation as well as the number of actin rings due to downregulation of osteoclast differentiation (Figure 3A,B). However, it did not inhibit the formation of resorption pits (Figure 3D), suggesting that purpurogallin primarily suppresses osteoclast differentiation, rather than osteoclast activity.

Osteoclast differentiation is initiated by the binding of RANKL expressed on osteoblasts to RANK on the osteoclast precursor cells. RANK activation in response to RANKL induces a cascade of phosphorylation events involving signaling molecules, including those belonging to MAPK and NF-κB pathways [29,30]. To understand the molecular mechanism(s) underlying the inhibitory action of purpurogallin, phosphorylation of molecules involved in the RANK signaling pathways was analyzed. Unexpectedly, the activation of RANKL-induced MAPKs, AKT, and NF-κB was not inhibited by purpurogallin (Figure 4A). Although the disruption of one of these signaling pathways induced by RANKL impairs osteoclast differentiation and function [31], purpurogallin showed no inhibitory effect on the RANK signaling pathways. Purpurogallin has been shown to exert anti-inflammatory activity via downregulation of NF-κB and MAPK [19]. MAPK and NF-κB signaling pathways are also activated by RANKL, but they were not downregulated by purpurogallin (Figure 4A). Both LPS and RANKL can activate MAPK and NF-κB, but purpurogallin only inhibits LPS-induced MAPK and NF-κB, suggesting that purpurogallin may preferentially target the LPS-induced signaling pathways, d leading to the activation of MAPK and NF-κB.

In an effort to further understand inhibitory mechanism of purpurogallin on osteoclast differentiation, we examined the expression of regulatory genes. Although the RANKL-induced activation of MAPK, AKT, and NF-κB was not inhibited by purpurogallin, the expression of c-Fos and NFATc1 was dramatically decreased (Figure 2B). c-Fos has been shown to be able to induce the expression of NFATc1 in osteoclasts [32]. In this context, the downregulation of c-Fos by purpurogallin at day 1 was followed by downregulation of NFATc1 at day 2–3 (Figure 2B). Therefore, downregulation of c-Fos and NFATc1 might be the primary mode of action of purpurogallin during osteoclast differentiation.

Blimp1, which is induced by RANKL through NFATc1 during osteoclastogenesis, functions as a transcriptional repressor of anti-osteoclastic genes such as *Irf8* [33]. IRF8 and Bcl6 also have shown to suppress the mRNA expression of Nfatc1 during osteoclastogenesis [34]. The expression of *Irf8* and *Bcl6* increased in the presence of purpurogallin (Figure 4B). Consistently, *Prdm1* was downregulated by purpurogallin (Figure 4B). These results demonstrated that purpurogallin impairs osteoclast differentiation in part by inducing the expression of negative regulators of osteoclast differentiation.

In summary, purpurogallin inhibits the differentiation of osteoclasts through downregulation of c-Fos-NFATc1-Blimp1 axis, leading to upregulation of negative regulators, Bcl6 and IRF8; however, it does not affect bone resorption in vitro.

## 4. Materials and Methods

### 4.1. Osteoclast Differentiation and TRAP Staining

All animal experiments were approved by the Committee on the Care and Use of Animals in Research at Kyungpook National University (with the approval number KNU 2017-0074 and approval date 5 June 2017), and were conducted in accordance with the guidelines for the care and use of laboratory animals.

Bone marrow cells isolated from 6- to 8-week-old C57/B6L mice were cultured in α-minimal essential medium (α-MEM) containing 10% fetal bovine serum (FBS) and M-CSF (30 ng/mL) for 3 days to obtain bone marrow macrophages (BMMs). Osteoclastogenesis was induced as described previously [35,36]. To examine its effect on osteoclast differentiation, purpurogallin (2,3,4,5-tetrahydroxy-5H-benzocycloahepten-5-one; Figure 1A) was enzymatically synthesized as described previously [27]. Purpurogallin (5 or 10 μM) were treated with M-CSF (10 ng/mL) and RANKL (20 ng/mL) for 4 days. The cells were fixed in 4% paraformaldehyde for 15 min and stained using the Acid Phosphatase, Leukocyte (TRAP) staining kit (Sigma-Aldrich, St. Louis, MO, USA). TRAP-positive multinucleated cells (MNCs) with more than 3 nuclei were counted as osteoclasts.

### 4.2. Cell Viability Assay

The viability of BMMs was determined using the 3-(4,5-dimethylthiazol-2-yl)-2,5-diphenyltetrazolium bromide (MTT) (Sigma-Aldrich, St. Louis, MO, USA). BMMs were cultured in a 96-well plate in the presence of M-CSF (10 ng/mL) with or without purpurogallin. After 3 days, MTT was added to each well, and the plate was incubated for 2 h. The absorbance was measured at 570 nm using a 96-well microplate reader (BioRad, Hercules, CA, USA).

### 4.3. Real-Time PCR

Total RNA was isolated using the TRI-solution (Bioscience, Seoul, Korea), and the isolated RNA was reverse transcribed using SuperScript II Reverse Transcriptase (Invitrogen, Carlsbad, CA, USA). Quantitative or real-time PCR was performed using the LightCycler 1.5 real-time PCR system (Roche Diagnostics, Basel, Switzerland) and SYBR Premix Ex Taq (Takara Bio Inc., Shiga, Japan). The primers used for quantitative PCR were as follows: *Acp5* or *TRAP*, 5′-TCCCCAATGCCCCATTC-3′ and 5′-CGGTTCTGGCGATCTCTTTG-3′; *Ctsk*, 5′-GGCTGTGGAGGCGGCTAT-3′ and 5′-AGAGTCAATGCCTCCGTTCTG-3′; *Dcstamp*, 5′-CTTCCGTGGGCCAGAAGTT-3′ and 5′-AGGCCAGTGCTGACTAGGATGA-3′; *Nfatc1*, 5′-ACCACCTTTCCGCAACCA-3′ and 5′-TTCCGTTTCCCGTTGCA-3′; *Irf8*, 5′-GATCGAACAGATCGACAGCA-3′ and 5′-AGCACAGCGTAACCTCGTCT-3′; *Bcl6*, 5′-ATGAGATTGCCCTGCATTTC-3′ and 5′-TTCTTCCAGTTGCAGGCTTT-3′; *Prdm1*, 5′-TTCTTGTGTGGTATTGTCGGGACTT-3′ and 5′-TTGGGGACACTCTTTGGGTAGAGTT-3′.

### 4.4. Western Blot Analysis

RIPA buffer containing protease and phosphatase inhibitors was used to prepare protein lysates. The concentration of protein lysates was measured using a BCA protein assay kit (Pierce Biotechnology, Rockford, IL, USA), and equivalent amounts of total protein (25 μg) were separated using 10% sodium dodecyl sulfate-polyacrylamide gel electrophoresis. Next, the separated proteins were transferred onto nitrocellulose membranes (Whatman, Florham Park, NJ, USA). The membranes were blocked using 3% non-fat dry milk in TBS-T [25 mM Tris-HCl (pH 7.4), 150 mM NaCl, and 0.1% Tween 20] for 1 h, and incubated overnight with primary antibodies at 4 °C, followed by incubation with the appropriate secondary antibodies for 1 h. Specific protein bands were detected by WesternBright ECL (Advansta, Menlo Park, CA, USA). Specific antibodies against phospho-p38, phospho-ERK, phospho-JNK, phospho-AKT, and phospho-IκBα were purchased from Cell Signaling Technology (Danvers, MA, USA). Monoclonal anti-β-actin was obtained from Sigma-Aldrich (St. Louis, MO, USA).

### 4.5. Resorption Pit Assay

To examine the bone resorbing activity of osteoclasts, mouse BMMs were seeded on bone slices (IDS Nordic, Herlev, Denmark), and cultured in an osteoclastogenic medium containing M-CSF (10 ng/mL) and RANKL (20 ng/mL) for 3 days. Next, the cells were treated with or without 10 μM purpurogallin for 2 days. All cells were removed from the bone slices, and resorption pits were visualized by staining with hematoxylin. The resorption pit area was analyzed using the i-Solution image analysis software (IMT i-Solution, Daejeon, Korea).

### 4.6. Immunofluorescence

BMMs seeded onto glass coverslips were cultured in an osteoclastogenic medium with vehicle or 10 μM purpurogallin for 4 days. The cells were fixed with 4% paraformaldehyde and permeabilized with 0.25% Triton X-100. The cells were incubated with the mouse anti-NFATc1 antibody, followed by the Alexa Fluor-488-conjugated secondary antibody. Actin rings and nuclei were stained with rhodamine-conjugated phalloidin (Cytoskeleton, Denver, CO, USA) and 4′,6-diamidino-2-phenylindole dihydrochloride (DAPI; Santa Cruz Biotechnology, Santa Cruz, CA, USA), respectively.

### 4.7. Statistical Analysis

All experiments were repeated three times, and data are presented as the mean ± standard deviation (SD). Data were analyzed using the two-tailed Student’s *t*-test or one-way analysis of variance (ANOVA) with Tukey’s multiple comparison post-hoc test. Values of *p* < 0.05 were considered to be statistically significant.
